# Continuous Wave Laser Nanowelding Process of Ag Nanowires on Flexible Polymer Substrates

**DOI:** 10.3390/nano11102511

**Published:** 2021-09-27

**Authors:** Li Xu, Wei-Chia Weng, Ying-Chin Yeh

**Affiliations:** Department of Mechanical Engineering, National Taiwan University, Taipei 10617, Taiwan; r04522309@ntu.edu.tw (W.-C.W.); r08522108@ntu.edu.tw (Y.-C.Y.)

**Keywords:** laser nanowelding, Ag nanowires, transparent conductive electrode, flexible device

## Abstract

In this paper we present the laser nanowelding process of silver nanowires (AgNWs) deposited on flexible polymer substrates by continuous wave (CW) lasers. CW lasers are cost-effective and can provide moderate power density, somewhere between nanosecond pulsed lasers and flash lamps, which is just enough to perform the nanowelding process efficiently and does not damage the nanowires on the polymer substrates. Here, an Nd:YAG CW laser (wavelength 532 nm) was used to perform the nanowelding of AgNWs on polyethylene terephthalate (PET) substrates. Key process parameters such as laser power, scan speed, and number of scans were studied and optimized, and mechanisms of observed phenomena are discussed. Our best result demonstrates a sheet resistance of 12 ohm/squ with a transmittance at λ = 550 nm of 92% for AgNW films on PET substrates. A transparent resistive heater was made, and IR pictures were taken to show the high uniformity of the CW laser nanowelded AgNW film. Our findings show that highly effective and efficient nanowelding can be achieved without the need of expensive pulse lasers or light sources, which may contribute to lower the cost of mass producing AgNWs on flexible substrates.

## 1. Introduction

Transparent conductive electrodes (TCEs) are one of the key components in optoelectronic devices such as solar cells [1], touch panel screens [2,3], smart displays [4], and wearable heaters [5]. Transparent conductive oxides (TCOs) such as indium tin oxide (ITO) are widely used as TCEs in these devices. However, conventional TCOs are not suitable for flexible devices [6] due to their mechanical properties as ceramic materials which can endure high compressive stress but are vulnerable to shear stress. As flexible devices require materials that are highly stretchable and bendable, new types of TCEs have been developed. Examples include carbon nanotubes (CNTs) [7], graphene [8,9,10], metal nanoparticle meshes [11,12], Ag patterns from particle-free reactive silver inks [13], and metal (Cu, Ag) nanowires (NWs) [14,15,16,17]. Kaempgen et al. [7] evaluated the transparency and conductivity of thin networks of carbon nanotubes sprayed onto glass or plastic substrates and achieved a transparency of 90% for visible light and a surface resistivity of 1 kΩ/squ. Eda et al. [8] reported a solution-based method to deposit reduced graphene oxide thin films and the sheet resistance could be tuned between 105 and 1011 Ω/squ with a transparency of 60~90%. Zheng et al. [9] synthesized single-walled carbon nanotubes (SWNTs) via a layer-by-layer Langmuir–Blodgett (L–B) assembly process and achieved sheet resistance in the range of 180~560 Ω/squ with optical transmittance between 77% and 86%. Hong et al. [12] fabricated a metallic grid transparent conductor on a flexible substrate using the selective laser sintering of metal nanoparticle ink, with transmittance >85% and sheet resistance of 30 Ω/squ. Han et al. [14] fabricated a CuNW-based percolation-network conductor by a laser nanoscale welding process. The sheet resistance of the conductor ranged from 18 to 5000 Ω/squ with a transparency between 70% and 90%. Metal nanowires such as gold, silver and copper nanowires showed promising results, achieving both low sheet resistance and high optical transparency. Among the metal materials of nanowires, gold is less desirable due to its high material cost; copper has lower cost, but not suitable for silicon-based devices because Pierret [18] illustrates that Cu as an impurity in Si provides deep level defects, which increases the recombination rate of the free carriers and thus compromises Si device performance. Ag material is favored because its electrical properties are compatible with Si and the cost of Ag material is moderate and acceptable. Manufacturers have been using screen-printable Ag paste to form front electrodes in silicon solar cells for more than a decade.

The optoelectronic properties of AgNW films not only depend on the length and diameter of the AgNWs, but also on the contact resistance between AgNWs. Hu et al. [19] showed that the resistances of Ag NWs with lengths ∼10 μm were ∼200–300 Ω for the individual wires and ∼450 Ω for wire−wire junctions. In order to improve the contact resistance between AgNWs, some processes have been developed to enhance the interconnection between nanowires in AgNWs. Tokuno et al. [20] improved the electrical conductivity of AgNW electrodes by mechanical pressing at 25 MPa for 5 s at room temperature, resulting in a low sheet resistance of 8.6 Ω/squ and a transmittance of 80.0%, equivalent to the properties of the AgNW electrodes heated at 200 °C. Hu et al. [19] developed a facile electrochemical annealing and pressing method to reduce the nanowire-to-nanowire junction resistance and achieved 20 Ω/squwith 80% specular transmittance in the visible spectral range. Spechler et al. [21] applied a 355 nm pulsed laser to anneal AgNW networks on glass substrates and achieved a sheet resistance of 3–6 Ω/squ with a transmittance of 55–70% at wavelength of 600 nm. Lee et al. [15] applied 532 nm nanosecond laser and continuous wave laser to process AgNWs on glass substrates. Their results showed that sheet resistance dropped to below 20 Ω/squ; however, damage or ablation of AgNWs at the laser scanning path was observed in SEM images of both laser types. Lee et al. [16] applied a xenon flash lamp to treat the AgNWs on polymer substrates and was able to improve the sheet resistance of AgNW films from 160 Ω/squ to 36.2 Ω/squ with transmittance of about 80% Ha et al. [24]. Used Ti:sapphire femtosecond laser (wavelength 800 nm) to process AgNWs on PET substrates, which improved the sheet resistance to about 25 Ω/squ and achieved transmittance of 94.3% at a wavelength of 550 nm. Hu et al. [22] also reported the femtosecond laser (wavelength 800 nm) welding of AgNWs on a PET substrate that yielded a sheet resistance of 16.1 Ω/sq and a transmittance of 91%.

Demonstration of the laser nanowelding AgNWs on polymer substrates has been done by either femtosecond laser, which minimizes the thermal effect; or by Xe flash lamp, which irradiates the surface with light of low power density, as shown in the summary table of the literature (Table 1). However, nanosecond pulsed laser and CW laser have been used to nanoweld AgNWs on glass substrates, but not on polymer substrates—probably because glass can endure high-power laser irradiation and has a higher damage threshold than polymer substrates. It is still unclear whether CW lasers can be used for polymer substrates without causing damage.

In this study, AgNWs were deposited on polymer substrates and a 532 nm CW laser was used for the laser nanowelding process. The advantage of using a CW laser is that it can avoid the high expense of femtosecond lasers and overcome the problems associated with flash lamps such as the unnecessary absorption of the wide spectrum of flash light and insufficient power density. The CW laser power density was carefully controlled to avoid damaging either the nanowires or the polymer substrate. The laser scanning system could provide fast process speeds and potentially be applied to large-scale production.

## 2. Experimental Setup

**Figure 1:**
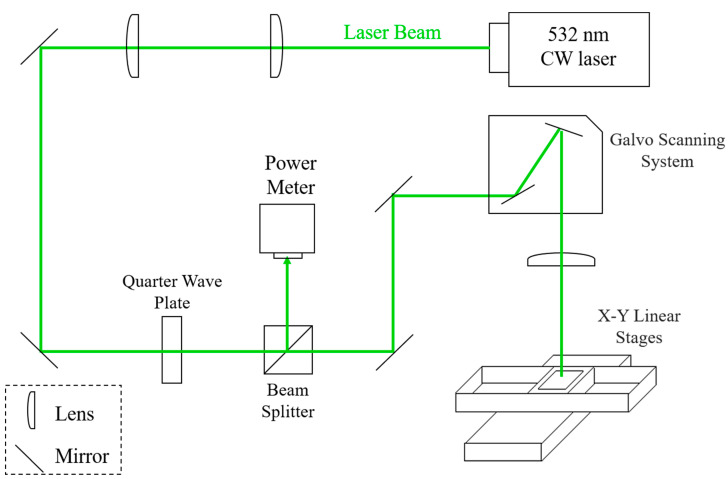
Schematic diagram of the laser scanning system.

A schematic diagram of the laser processing system is shown in Figure 1. The system consisted of a solid-state Nd:YAG continuous wave (CW) laser (wavelength λ = 532 nm), plano-convex lens, mirrors, quarter-wave plate, beam splitter and a galvo scanner system. Two focal lenses were used to collimate the laser beam and expand the beam size. The quarter-wave plate was used to transform the laser light from linear polarization to circular polarization to avoid the influence of any coupling effect of the polarization direction and the longitudinal direction of AgNWs. The beam splitter was used to divert 10% of the laser light, and the laser output power was monitored by a power meter. The galvanometer scanner system was employed to scan the laser beam at the designed path speed and pattern. The final focal lens focused the laser beam on the sample sitting on the X–Y linear moving stage. The beam spot size at the focal point was measured to be about 50 μm at full width half maximum (FWHM). The laser power ranged from 200 mW to 1300 mW, and the scan speed (V) varied from 50 to 200 mm/s with a line-to-line overlap of 50%. The processed areas on the sample were then stitched together by moving the X–Y linear stage for large-area processing.

Silver nanowires, provided by Conjutek Ltd. (New Taipei City, Taiwan), were synthesized with the polyol method [7]. The average dimensions of the silver nanowires were 90 nm in diameter and 20 μm in length. The silver nanowires were transferred onto 50 μm-thick polyethylene terephthalate (PET) films provided by Nan Ya Plastics (Taipei, Taiwan) by vacuum filtration [25] to make the AgNWs randomly and evenly distributed. A four-point probe was used to measure the sheet resistance, and a UV-VIS spectrometer equipped with an integrating sphere and tungsten halogen lamps was used as a light source to measure transmittance. The morphology of the AgNWs after laser processing was inspected using a field-emission scanning electron microscope (NOVA NANO SEM 450).

## 3. Results and Discussion

### 3.1. Correlation of Sheet Resistance and Transmittance

**Figure 2:**
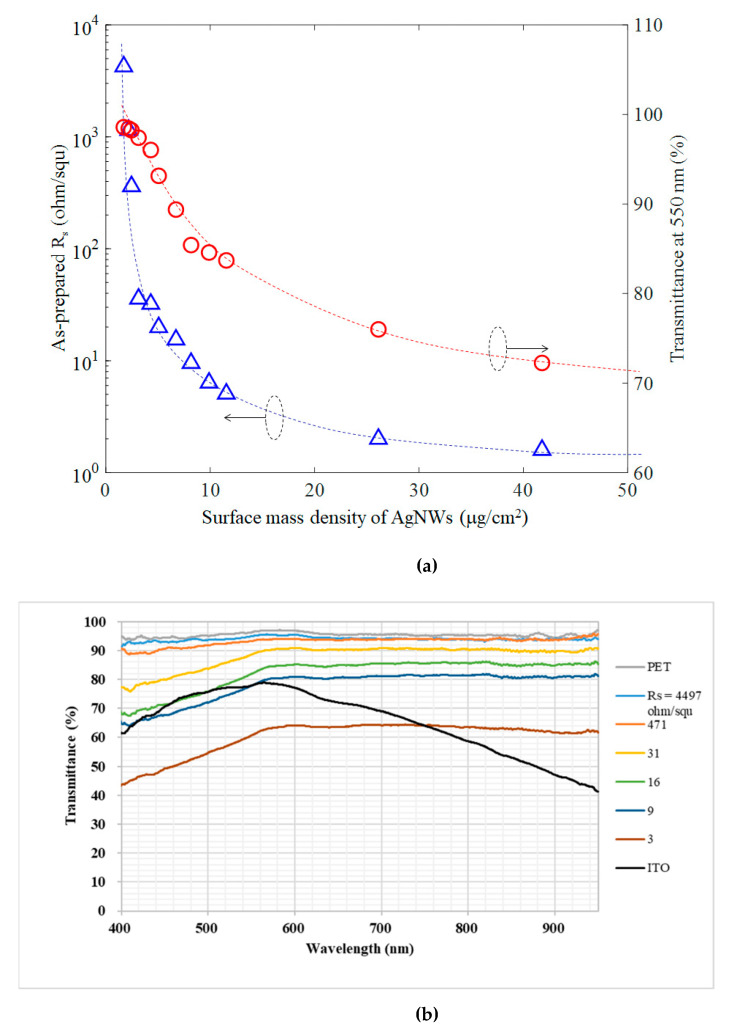
(**a**) Sheet resistance and transmittance (at 550 nm) as a function of the surface mass density of as-prepared AgNWs films; (**b**) Comparison of transmission curves of PET films, as-prepared AgNWs with different sheet resistance on PET and ITO on PET substrate.

The sheet resistance and optical transmittance of AgNW films are closely correlated and both dependent on the amount of AgNWs deposited on the substrate. We defined a parameter called the surface mass density (μg/cm^2^) (i.e., the mass of AgNWs (μg) per unit area (cm^2^)) to quantify the amount of AgNWs deposited on the substrate. A higher surface mass density of AgNWs resulted in a lower sheet resistance, as shown in Figure 2a. When the surface mass density was in the low range of less than about 10 μg/cm^2^, the sheet resistance increased dramatically with decreasing surface mass density. This was probably because the amount of contact points between AgNWs was significantly reduced when the number of AgNWs per unit area was low, that is, when the AgNWs were sparsely distributed, providing fewer paths for electron transport. When the surface mass density was high, the number of contacts increased dramatically, which provided many more paths for efficient electron transport. On the other hand, a high surface density resulted in less light transmission, as shown in Figure 2a (right *y*-axis), due to the agglomeration of Ag nanowires at high mass density, resulting in the blockage of light. We took the transmittance at the wavelength of 550 nm in the visible light range as a representative for comparison. The transmission data in the full wavelength range of visible light is shown in Figure 2b with various AgNW sheet resistances as well as bare PET substrate and ITO film on PET substrate.

### 3.2. Study of Laser Processing Parameters 

#### 3.2.1. Effect of Laser Power

**Figure 3:**
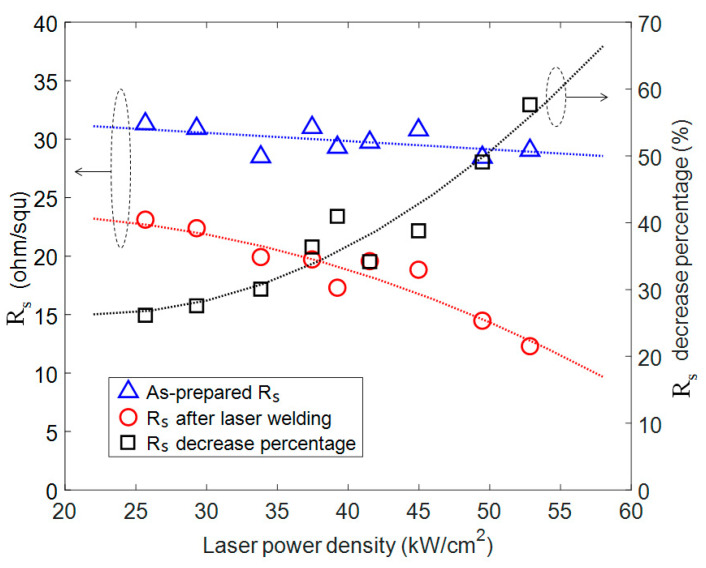
Sheet resistance change as function of laser power density. Blue triangles are the as-prepared R_s_ and the red circles are the R_s_ after laser welding of the same sample. The dark squares are the percentage decrease of R_s_ of each sample. A fixed scan speed of 100 mm/s and a single time scan were used.

The effect of laser power density on the sheet resistance is shown in Figure 3, with the other parameters fixed (scan speed 100 mm/s and single time scan). The blue triangles represent the sheet resistance (R_s_) of as-prepared samples and the red circles are the sheet resistance of the same sample after the laser nanowelding process. As the laser power increased from 26 to 53 kW/cm^2^, R_s_ dropped from around 30 ohm/squ to the range of 23–12 ohm/squ. The percentage decrease of R_s_ (the right axis in Figure 3) was used to evaluate the change in R_s_ to eliminate the variation of as-prepared R_s_; it was defined as the amount of R_s_ decrease divided by the R_s_ of as-prepared AgNWs films. The percentage decrease of R_s_ increased from 26% to 58% as laser power increased from 26 to 53 kW/cm^2^. A higher laser power caused more energy absorption at the intersection of AgNWs; as a result, the volume of melted Ag material increased and there was stronger bonding between AgNWs and improved electrical conductance of the AgNWs film. When the laser power was increased beyond 53 kW/cm^2^, PET substrate damage was observed. The transmittance of all samples did not show any significant change before and after the laser nanowelding process.

**Figure 4:**
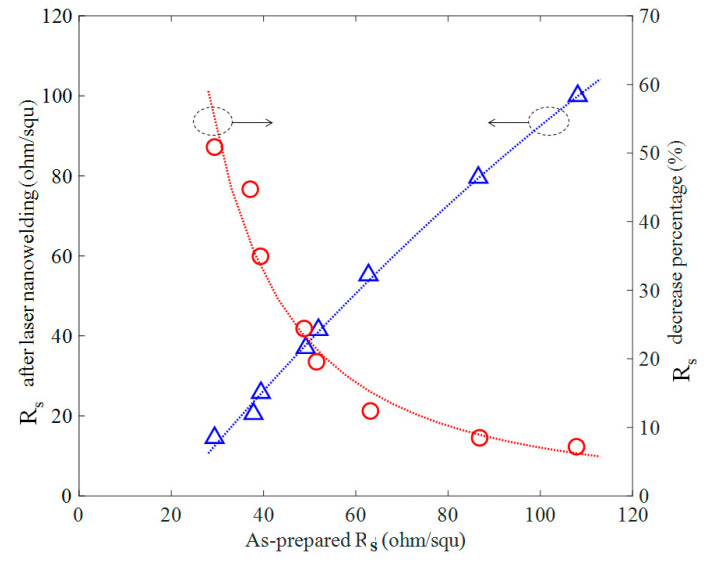
Sheet resistance after the laser nanowelding (left axis) and R_s_ decrease percentage (right axis) as function of the as-prepared R_s_.

CW laser nanowelding process was more effective on the AgNW films with lower as-prepared Rs as shown in Figure 4. Samples with different as-prepared Rs experienced the same laser nanowelding process (laser power of 46 kW/cm^2^, scan speed of 100 mm/s, single scan). It was observed that the Rs decrease percentage was less significant when as-prepared Rs was high. It was probably because the samples with higher as-prepared Rs corresponded to less amount of AgNWs per unit area, so that fewer number of contacts among AgNWs were available to be laser nanowelded, and thus the laser nano-welding process was less effective on high Rs samples.

#### 3.2.2. Effect of Laser Scan Speed

**Figure 5:**
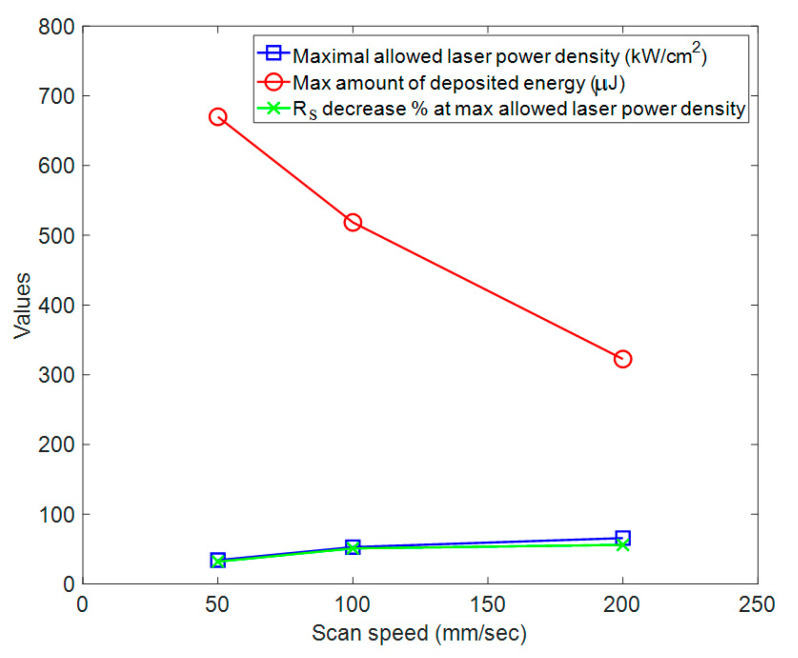
Effect of scan speed on the laser nanowelding process. As the scan speed increased, maximum allowed laser power density (blue) increased. The total amount of energy (red) decreased as the dwell time at higher scan speed was shorter. The percentage decrease of R_s_ (green) was more strongly correlated with laser power density rather than the total amount of energy. All data are based on a single time scan.

Laser scan speed (V) influenced the time the laser beam stayed at each point of the sample (i.e., dwell time (t)). Dwell time was calculated as t = D/V, where D is the diameter of the beam. We studied three scan speeds of 50, 100 and 200 mm/s, which corresponded to dwell times of 1000 μs, 500 μs and 250 μs, respectively. It was observed that the PET damage threshold varied at different scan speeds. Figure 5 shows the maximal laser power density that could be applied without damaging the PET substrate, the amount of energy deposited at each point and the resulting R_s_ percent decrease at these conditions.

It was observed in all experimental conditions that the damage threshold of laser power density for the PET film was lower than that for AgNWs, which determined the maximal allowed laser power density. Figure 5 shows that as the scan speed increased, the maximal allowed laser power density increased, but the total amount of energy decreased because the dwell time at the higher scan speed was shorter. The percentage decrease of R_s_ of AgNWs was more strongly correlated with the applied laser power density than the amount of the energy deposited on the AgNWs, suggesting that the instant high temperature increase resulting from high-intensity laser light absorption induced the melting of Ag material at the intersections, while low-intensity laser annealing for a longer time could not achieve these results.

The minimum required dwell time reduced as the laser power density increased. As the limit of the laser power of our system was reached at 1.3 W, and the minimum required dwell time was not obtained in the experiments. Theoretically, the minimum required dwell time can be obtained as the laser power increases to the point when AgNWs start to melt, causing damage (balling) of the AgNWs.

#### 3.2.3. Effect of the Number of Scans

**Figure 6:**
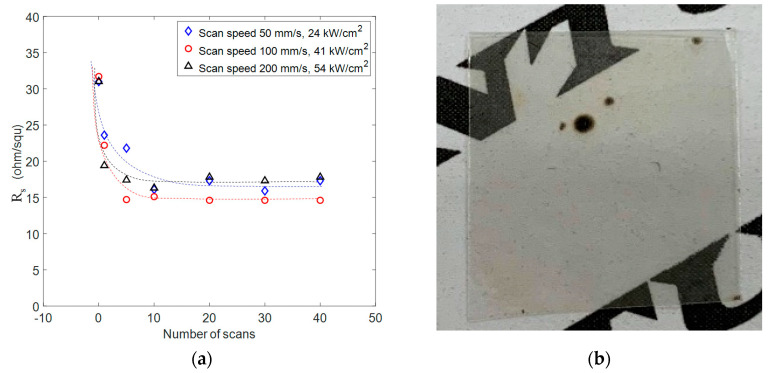
(**a**) Sheet resistance decreased then kept constant as number of scans increased at various scan speed. (**b**) A picture of damaged AgNWs film on PET. The dark spots were the damaged part of PET film at high laser power density.

The number of scans used in the laser process also influenced the sheet resistance reduction to a certain extent. Figure 6 shows the R_s_ values at various scan speeds and scan numbers. We observed that more scans resulted in smaller R_s_ values; however, at a scan number of about 10, the percentage decrease of R_s_ saturated and R_s_ did not change further with more scans. This is probably because the intersections of AgNWs had all been welded together and further laser illumination did not change the properties of the AgNW films. Our best result was 12 ohm/squ, with a high transmittance of 92% at the condition of 100 mm/s, single scan and a laser power density of 53 kW/cm^2^. We observed that substrate damage occurred at random spots on the sample. This was probably because the high temperature of AgNWs resulting from strong laser absorption caused local carbonization of the polymer substrate, which enhanced direct absorption of the laser light and further damaged the carbonized spot in the repeated scans. The lower damage threshold in multiple scans was the reason that the laser power in Figure 6a was set lower than the damage threshold in the case of a single scan, as shown in Figure 5.

### 3.3. Morphology of Laser Processed AgNWs

**Figure 7:**
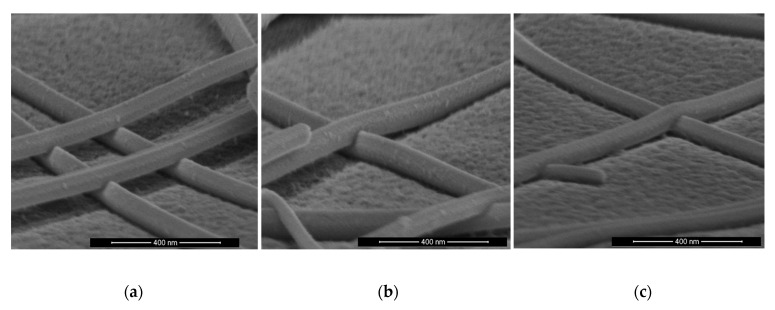

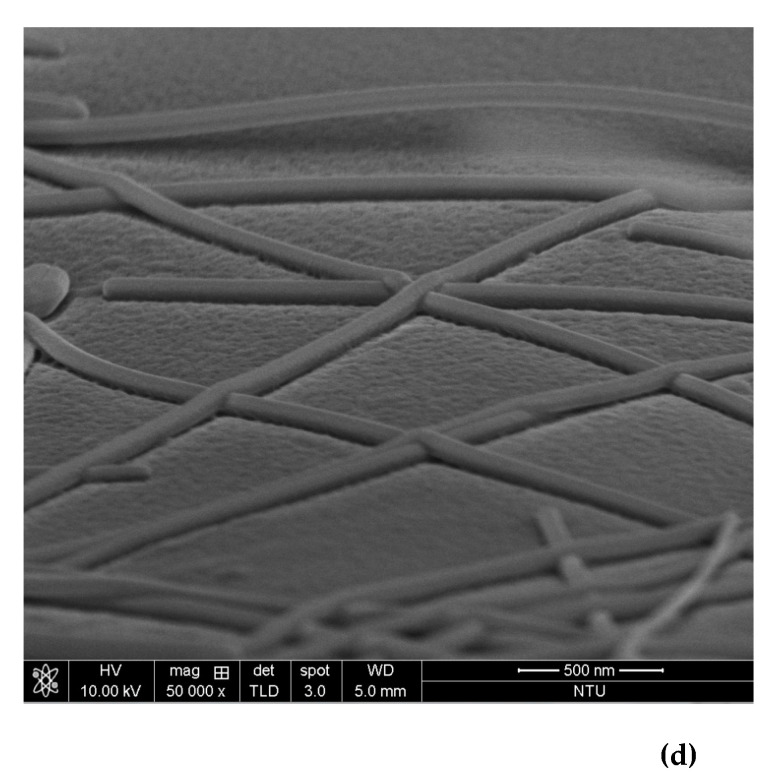
SEM images of AgNWs on PET substrate after laser nanowelding at laser power of (**a**) 28 kW/cm^2^, (**b**) 40 kW/cm^2^ and (**c**) 50 kW/cm^2^ with the fixed scan speed of 100 mm/s and 5 scans; (**d**) a larger-scale SEM image of the sample in (**c**) showing no ablation or balling of AgNWs.

High-resolution SEM images were taken to inspect the morphology of AgNWs after the laser nanowelding process, as shown in Figure 7. As the laser power increased from 28 to 50 kW/cm^2^ (from Figure 7a to Figure 7c), more Ag material at the contact was observed to embed into each other, suggesting that more Ag material melted and welded together upon higher-power laser illumination. The welded parts of AgNWs were strongly bonded and provided efficient electron transportation paths between AgNWs, resulting in lowered sheet resistance ranging from 21.3 to 14.4 ohm/squ with the same as-prepared sheet resistance of 30 ohm/squ. Figure 7d is a close-up view of the sample in Figure 7c, and no ablation or balling of the nanowires can be observed.

### 3.4. Bending and Stretching Tests

**Figure 8:**
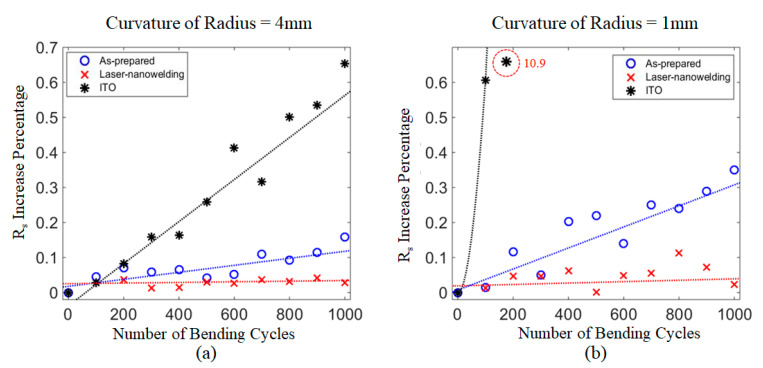
Sheet resistance (Rs) increase percentage of bending tests at the radius of curvature of (**a**) 4 mm and (**b**) 1 mm.

The mechanical properties of the AgNW films on PET substrate were tested and compared with ITO film on PET by bending the films with different curvatures and stretching the films with different strains. Figure 8 shows the results of the bending tests at the radii of curvature of 4 mm and 1 mm. At the 4 mm radius of curvature (Figure 8a) the sheet resistance of ITO and as-prepared AgNWs films increased by 65% and 16%, respectively, after 1000 bending cycles, whereas the value of AgNW films after the laser nanowelding process remained largely unchanged. At the radius of curvature of 1 mm (Figure 8b) the sheet resistance of ITO films increased dramatically by 10.9 times after 200 cycles, followed by cracking, and the value of the as-prepared AgNW films increased by 35% after 1000 cycles. By contrast, the AgNW films treated with the laser nanowelding process showed almost no change, with less than 10% variation or instability after being subjected to a 1 mm radius of curvature bending. The excellent performance of AgNW films after laser nanowelding may be attributable to the strong bonding at the intersection of the AgNWs, which made the randomly distributed AgNW films into strongly linked networks.

**Figure 9:**
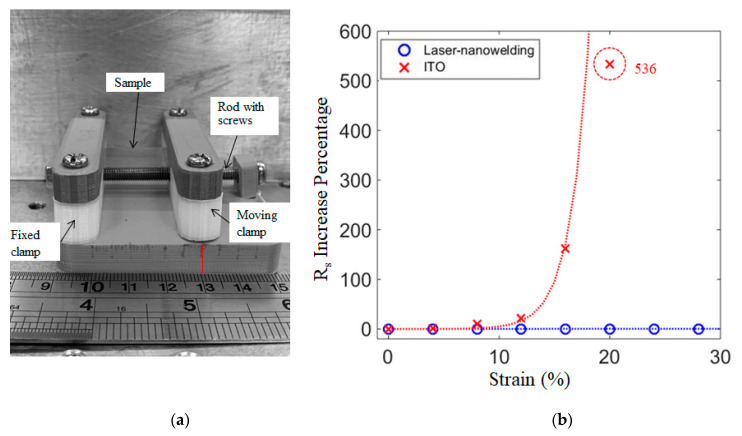
Stain tests of AgNWs on PET films. (**a**) Stain test device; (**b**) Sheet resistance change in stretching tests.particles with different surface functional groups as a function of medium conductivity. (**b**) The case for 3-μm particles.

In the stretching tests, AgNWs on PET films were mounted on the strain test device with a fixed clamp and a moving clamp (Figure 9a), and a rod with screws was used to adjust the distance between the clamps. Samples were stretched to a certain strain and then relaxed, and the sheet resistance of the films after relaxation was measured. The results of the stretching tests (Figure 9b) show that sheet resistance of ITO films increased dramatically when the strain was beyond 12% and increased by 536 times at 20% strain, with some parts of the film cracking. AgNWs films subjected to the laser nanowelding process showed almost no change as the strain increased to 30%. This demonstrates that the nanowelding process significantly improved the stretchability of the AgNWs, and is suitable to be applied on flexible devices.

### 3.5. Fabrication of a Transparent Resistive Heater

**Figure 10:**
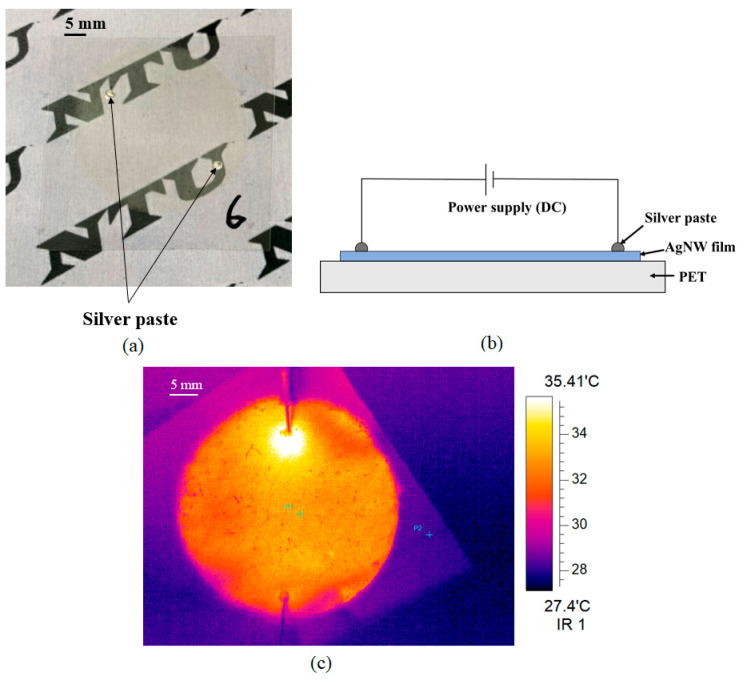
Resistive heater built with CW laser nanowelded AgNW films. (**a**) AgNW film (circle) on PET substrate (square); (**b**) schematic diagram of the measurement setup; (**c**) infrared picture of AgNW film under 5 V voltage.

The CW laser-welded AgNW film was made into a transparent resistive heater to demonstrate its good uniformity of electrical conductance after the laser welding process. The AgNW film deposited on the polymer substrate was subject to the CW laser nanowelding process, and silver paste was applied at the edge of the AgNW film as contact for measurement, which is shown as small dots in Figure 10a. A schematic diagram of the measurement setup is shown in Figure 10b, in which a 5 V DC power was applied between two silver paste contacts. As steady state was reached, the surface temperature was measured with an infrared (IR) camera provided by Ching Hsing Computer-Tech Ltd. (Taipei, Taiwan) (model: IRM-P384G3-20-16 um). The temperature of the AgNW films at point 1 (P1) was 33.34 °C, while that of the bare PET film at point 2 (P2) was 28.15 °C, as shown in Figure 10c. The IR image shows that the resistive heater worked well with good uniformity and the bright spot at the contact point was due to the current-crowding phenomenon. A good uniformity of the AgNWs and the laser scan process is important if the technology is to be applied to large-scale production.

## 4. Conclusions

In this study we demonstrate that the nanowelding process using cost-effective CW lasers can significantly enhance the electrical and mechanical characteristics of Ag nanowire films on flexible substrates such as PET. However, the optimization of key laser parameters is required in order to achieve good results without damaging the AgNWs and substrate. Specifically, we investigated and optimized laser power density, scan speed and scan times, and highlight the following key observations. First, the laser power damage threshold for PET films was lower than that for AgNWs at all experimental conditions. Second, the damage threshold increased with the scan speed. A higher scan speed allows a higher laser power density, but the total amount of energy deposited at each point decreases as the dwell time decreases at higher scan speeds. Third, the reduction of sheet resistance was more strongly related to the laser power density than the total amount of deposited energy. Sheet resistance as low as 12 ohm/squ was achieved with a high transmittance of 92% (at 550 nm) at the process conditions of laser power density of 53 kW/cm^2^, 100 mm/s scan speed and single scan. SEM images confirmed that AgNWs physically welded at the junctions and no balling or ablation of AgNWs were observed. The laser-nanowelded AgNW films also exhibited excellent flexibility and stretchability, making them suitable for application in flexible devices. Finally, a transparent resistive heater was successfully made with a laser nanowelded AgNW film showing high uniformity the electrical conductance of the films.

## Figures and Tables

**Table 1:** Summary of laser nanowelding process of AgNWs.

Substrate	LASER TYPE	Pulse Width	Wavelength (nm)	Fluence (mJ/cm^2^)	Power Density (kW/cm^2^)	R_s_ (Ω/squ)	Transmittance	Morphology of AgNWs	Ref.
Glass	ns pulsed laser	3 ns	355	30~50	---	3~6	55~70% (at 600 nm)	Balling	Spechler 2012 [21]
	ns pulsed laser	5 ns	532	17.4~37.7	---	<10	95%	Balling	Lee 2017 [15]
	CW laser	---	532	---	500	<20	96%	Balling and ablation	Lee 2017 [15]
Silicon Wafer	ns pulsed laser	27 ns	355	23.5	---	~2.5	~82% (at 26% AgNWs area coverage)	Balling	Spechler 2015 [23]
Polymer	fs pulsed laser	50 fs	800	30~120	---	25	94.3% (at 633 nm)	Intact	Ha 2016 [24]
	fs pulsed laser	120 fs	800	0.2~2 × 10^−3^	---	16.1	91% (at 550 nm)	Intact	Hu 2019 [22]
	Xe flash light	---	400~1000	4000~7000	(not specified)	36.2	80% (at 550 nm)	Intact	Lee 2018 [16]
	CW laser	---	532	---	53	12	92% (at 550 nm)	Intact	This work

## Data Availability

Raw and processed data available upon request.
